# Editorial: Deciphering the role of signature genes in cancer prognosis and therapy resistance

**DOI:** 10.3389/fonc.2025.1591256

**Published:** 2025-04-08

**Authors:** Dalila L. Zanette, Manoj K. Pandey, Jawed A. Siddiqui

**Affiliations:** ^1^ Carlos Chagas Institute, Oswaldo Cruz Foundation (Fiocruz/Paraná), Rio de Janeiro, Brazil; ^2^ Department of Biomedical Sciences, Cooper Medical School of Rowan University, Camden, NJ, United States; ^3^ Department of Cell and Molecular Biology, University of Mississippi Medical Center, Jackson, MS, United States; ^4^ Cancer Center Research Institute, University of Mississippi Medical Center, Jackson, MS, United States

**Keywords:** cancer, therapy resistance, tumor microenvironment, immune checkpoint inhibitor (ICI), metastasis

Despite decades of research on cancer and its effects on patient care, it continues to pose a significant health challenge. The characterization of the multi-faceted disease is significantly influenced by tumor angiogenesis and therapy response, which are driven by genetic and epigenetic alterations. Preventive medicine, therapy design, and personalized care are interconnected concepts that relate to the variability in molecular determinants of cancer or gene signatures, which affect prognosis and treatment strategies. Gene signatures facilitate the recognition and functional characterization of entities through high-throughput genome and transcriptome analysis, RNA sequencing methodologies, single-cell omics investigations, and microarray profiling. The altered expression profile of various gene signatures indicates dysregulated cellular processes. The irregular expression of the gene-signature profile is directly linked to enhanced cell proliferation, immune system evasion, disruption of apoptotic pathways, epithelial to mesenchymal transition, alterations in the tumor microenvironment, and therapy resistance.

This Research Topic, titled *“Deciphering the Role of Signature Genes in Cancer Prognosis and Therapy Resistance,”* focuses on recent advancements in understanding the genetic signatures that influence cancer progression, as well as their contributions to therapy resistance and metastatic potential. This Research Topic comprises nine articles: seven original research articles (Liu et al., Mao et al., Li et al., Wang et al., Benevides et al., Terrones et al., and Sonnemann et al.), one review article (Wu et al.), and one mini review (Coelho et al.).

The application of immune checkpoint inhibitors (ICIs) has gained widespread acceptance for the treatment of patients with advanced Hepatocellular carcinoma (HCC). Liu et al. conducted a study involving 54 patients with hepatocellular carcinoma (HCC), utilizing SVM-RFE modeling on six immune-related genes (IRGs): CMTM7, HDAC1, HRAS, PSMD1, RAET1E, and TXLNA. They developed a novel approach to identify overall survival and the impact of immunotherapy in HCC [1]. A study by Mao et al. on HCC identified six survival-related genes (BMI1, CCR3, CDC25C, CFL1, LDHA, RAC1) associated with the CCL18 signaling pathway. Additionally, enhanced cell proliferation, migration, and stemness were reported in response to the overexpression of these six genes [2].

Gastric cancer (GC) is recognized as a prevalent malignancy, and conventional treatment methods are insufficient for achieving favorable patient prognoses. Li et al. conducted a study utilizing the TCGA database to develop a novel prognostic system for gastric cancer patients. The authors emphasized a notable prognostic distinction between high and low-risk GC groups, indicating that risk scoring has a more substantial impact on prognosis than tumor stage identification in GC. This study is the inaugural effort in developing a model that emphasizes the significance of platelet-related genes in gastric cancer progression, metastasis, and resistance to therapy [3]. A study by Wang et al. developed a predictive model for glycosylation-related genes to elucidate the broader implications of immunotherapy for gastric cancer. GLT8D2 has been identified as a significant prognostic marker with a robust correlation to Tumor Infiltrating Lymphocytes (TILs), encompassing CD8+ T cells, CD4+ T cells, Treg cells, B cells, neutrophils, dendritic cells (DCs), natural killer (NK) cells, and monocytes, especially macrophages in GC [4].

The expression of long noncoding RNAs (lncRNAs), particularly HOTAIR, is well-documented in solid tumors. A study by Benevides et al. investigated the expression of HOTAIR and PTGS2 in 87 patients with Chronic Myeloid Leukemia (CML). Samples of CML exhibit significant downregulation in the expression of these two genes. Additionally, they emphasized the inverse correlation between BCR: ABL1 expression and HOTAIR and PTGS2 in CML patients undergoing imatinib treatment, highlighting the possible regulatory interactions between these factors in the context of the CML therapy [5].

ROS1+ non-small cell lung cancer (NSCLC) represents a molecular subgroup comprising approximately 2% of newly diagnosed lung cancers each year. Terrones et al. conducted an analysis of the transcriptomic characteristics of ROS1+ NSCLC samples utilizing data from The Cancer Genome Atlas (TCGA) and the Gene Expression Omnibus (GEO) databases [6]. The authors observed an upregulation in pathways related to nucleotide synthesis and cell adhesion. The downregulation of NOTCH1 correlates with the reduced expression of PD-L1 in ROS1+ NSCLC. This study highlights the significance of nucleotide synthesis and cell adhesion in elucidating the pathophysiology of ROS1+ NSCLC [6].

A study by Sonnemann et al. investigated the transcriptional activator Vestigial-like 1 (VGLL1), elucidating its cellular function and downstream targets in placental, breast, and pancreatic cancer cells [7]. The authors conducted ChIP-seq analysis to identify eight transcription factors with VGLL1-binding motifs. Additionally, increased expression of VGLL1 was associated with enhanced cell invasion and proliferation, highlighting its potential as a key player in cancer progression [7].

The complexity of prostate cancer (PCa) is significantly influenced by the genetic signature of the individual patient. Wu et al. summarize that the progression of prostate cancer (PCa) and its eventual advancement to therapy resistance and lethality is driven by the genetic interactions among the androgen receptor (AR), retinoblastoma (Rb), PTEN, WNT, p53, and MYC. This review elaborates on the functional and therapeutic potential of these key genes, which hold promise for future breakthroughs and the development of novel drugs to tailor treatment options for PCa [8].

Coelho et al. have described urinary mRNA-based biomarkers as potential tools for studying aggressiveness in non-muscle invasive bladder cancer (NMIBC), a type characterized by high proliferation and recurrence [9]. The presence of both shared (IGF2, ANAXA10, CRH) and exclusive (ABC1 and UPK1B) mRNA-based biomarkers for NMIBC is proposed to enhance prognosis and inform targeted therapy design for this type of bladder cancer [9].

This Research Topic will be an essential resource for researchers investigating the intricacies of hallmark genes in cancer prognosis and therapeutic resistance. We seek to offer an extensive understanding of how these genetic fingerprints affect disease development, treatment response, and resistance mechanisms by integrating advanced studies and expert insights. We anticipate that the insights presented in this Research Topic will augment existing knowledge and stimulate innovative approaches for advancing cancer diagnostics, prognostication, and therapeutic interventions.

